# Volatile Organic Compounds (VOCs) in Neurodegenerative Diseases (NDDs): Diagnostic Potential and Analytical Approaches

**DOI:** 10.3390/molecules30194028

**Published:** 2025-10-09

**Authors:** Jolanda Palmisani, Antonella Maria Aresta, Viviana Vergaro, Giovanna Mancini, Miriana Cosma Mazzola, Marirosa Rosaria Nisi, Lucia Pastore, Valentina Pizzillo, Nicoletta De Vietro, Chiara Boncristiani, Giuseppe Ciccarella, Carlo Zambonin, Gianluigi de Gennaro, Alessia Di Gilio

**Affiliations:** 1 Department of Biosciences, Biotechnology and Environment, University of Bari, Via Edoardo Orabona, 70125 Bari, Italy; jolanda.palmisani@uniba.it (J.P.); marirosa.nisi@uniba.it (M.R.N.); nicoletta.devietro@uniba.it (N.D.V.); gianluigi.degennaro@uniba.it (G.d.G.); alessia.digilio@uniba.it (A.D.G.); 2 Department of Experimental Medicine, University of Salento c/o College Isufi, Centro Ecoteckne, Via Monteroni, 73100 Lecce, Italy; 3 Department of Biological and Environmental Sciences and Technologies (DiSTeBA), University of Salento, Via Monteroni, 73100 Lecce, Italy; giuseppe.ciccarella@unisalento.it

**Keywords:** Alzheimer’s disease, Parkinson’s disease, neurodegenerative diseases (NDDs), environmental risk factors, volatile organic compounds (VOCs), early-diagnosis approaches, biomarkers

## Abstract

Neurodegenerative diseases (NDDs) are a group of progressive diseases affecting neuronal cells in specific areas of the brain, causing cognitive decline and movement impairment. Nowadays, NDDs play a significant role in the global burden of disease, and their incidence is increasing, particularly due to population aging. NDD onset is multi-factorial; based on the current knowledge, genetic, environmental, and cellular factors are believed to contribute to their occurrence and progression. Taking into account that at an early stage, the symptoms are not clearly defined, and diagnosis may be delayed, the development of innovative and non-invasive methodological approaches for early diagnosis of NDDs is strategic for timely and tailored disease management, as well as for the overall improvement of patients’ quality of life. The present review aims to provide, in the first part, an overview based on the current level of knowledge on the environmental risk factors that can explicate a role in the onset of the most common NDDs and on the main pathogenic mechanisms involved in disease initiation and progression. The second part aims to define the current state of the art regarding the significance of Volatile Organic Compounds (VOCs) in the volatome of different human biological matrices (exhaled breath, feces, and skin sebum) as candidate biomarkers of specific NDDs, with the aim of developing non-invasive diagnostic approaches for the early diagnosis and personalized management of the patients. A critical synthesis and discussion on the applied methodological approaches and on the relevant outcomes obtained across the studies is reported.

## 1. Introduction

### 1.1. Neurodegenerative Diseases

Neurodegenerative diseases (NDDs) are a heterogeneous group of neurological disorders characterized by the progressive loss and often irreversible damage of neuronal cells in specific brain areas [1]. These conditions are typically marked by a gradual decline in neurological function, including demyelination, dendritic retraction, and neuronal loss, which collectively contribute to impairments in cognition (e.g., dementia) and motor control (e.g., ataxia). NDDs include Alzheimer’s disease (AD), frontotemporal dementia (FTD), amyotrophic lateral sclerosis (ALS), Lewy bodies dementia (LBD), Parkinson’s disease (PD), Huntington’s disease (HD), Friedreich’s ataxia (FRDA) and prion disease. Although age is the main risk factor contributing to the onset of NDDs, recent studies revealed an increased NDD risk associated with genetic and environmental factors [2,3,4,5]. In fact, although NDDs commonly have a late onset, recent evidence highlighted that NDDs can also impact early-life cognitive function, behavior, and brain structure and function [6,7]. The severity of symptoms increases as the disease progresses, resulting over the time in individuals being unable to live independently [7]. Since the first symptoms of NDDs appear only after neuronal death, the early detection of these diseases is currently impossible [8]. In fact, diagnosis of NDDs is based on clinical evaluation and imaging investigation such as positron emission tomography (PET), CT (Computer Tomography), or MRI (Magnetic Resonance Imaging). These techniques provide information about the position and dimension of deterioration in the central nervous system (CNS) tissue, thus offering an overview of the disease progression when it is already at an advanced stage. Moreover, the application of such imaging techniques presents several limitations, including high costs and health risks for patients [9]. On the other hand, to date, diagnostic methods based on biomarker quantification on biofluid such as cerebrospinal fluid still encounter challenges due to the low detectability of specific biomarkers and the invasiveness of the procedure.

Considering that neurodegenerative diseases represent one of the main causes of public health concerns, affecting almost 180 million people worldwide, the development of a method that is non-invasive, safe to apply, rapid to administer, and able to provide early diagnosis of these pathologies is a priority [10]. For this purpose, understanding of the disease pathways and pathophysiological features underlying the initiation of NDDs, as well as all the susceptibility factors or risk factors affecting NDD incidence and progression, is crucial. A common pathological thread among various neurodegenerative disorders is the presence of oxidative and nitrosative stress (OS, NS) and neuroinflammation [11]. 

OS arises when the production of reactive oxygen species (ROS) surpasses the capacity of the body’s antioxidant defenses, leading to cellular damage. Although ROS are natural by-products of aerobic metabolism, their excessive accumulation due to either increased production or impaired detoxification can initiate and propagate neurodegenerative processes. Similarly to OS, NS is characterized by the overproduction of reactive nitrogen species (RNS).

Emerging evidence suggests that both OS and NS trigger multiple deleterious molecular pathways, which interact in complex and not yet fully understood ways. It is this intricate interplay, rather than a single isolated mechanism, that likely drives the progression of neurodegeneration [12,13,14]. 

In parallel, neuroinflammation, a typically protective immune response, can become maladaptive when prolonged or dysregulated. Inflammatory processes within the central or peripheral nervous system may contribute to neuronal injury and are increasingly recognized as key contributors to the onset and progression of chronic neurodegenerative diseases [15,16,17]. 

Nowadays, mutations in genes such as *SOD1* or disruptions in glutathione homeostasis, as well as environmental exposure to air pollution, have been implicated in exacerbating oxidative damage and neuroinflammation.

The identification of biomarkers linked to the processes in biofluids that are easy to analyze, as well as the environmental factors that are potentially involved in the onset of NDDs, could help to develop a useful, non-invasive, and easy-to-use approach for interception of the neurodegeneration development and, thus, for the early diagnosis of NDDs [10]. 

Therefore, this review aims to highlight the environmental risk factors linked to NDDs, the pathogenic mechanisms involved in the initiation and progression of the pathologies, and potential biomarkers linked to them. Bearing this in mind, we evaluate the role of VOCs detectable in the main human biofluids as potential biomarkers for non-invasive diagnosis of NDDs. VOCs are metabolites that are continuously released in the human body as intermediates or products of cellular metabolic pathways, and their composition in biofluids is affected by both qualitative and quantitative changes depending on cellular pathophysiological conditions [18]. 

### 1.2. Environmental Risk Factors for NDD Onset

The World Health Organization lists air pollution as one of the top five risk factors contributing to non-communicable chronic disease onset, along with tobacco and alcohol consumption, unhealthy diet, and sedentary lifestyle [19]. 

People are exposed to complex mixtures of chemicals on a daily basis by consuming contaminated food and drink, using consumer products, and breathing polluted air [20]. Indoor and outdoor air impairment in terms of chemical, physical, or biological pollutants negatively affect human health, the ecosystem, and the climate. More specifically, long-term exposure to chemical air pollutants can increase the risk of respiratory and cardiovascular diseases and may contribute to the onset of CNS diseases, where environmental factors may trigger or exacerbate neurodegenerative processes [21]. However, due to the time delay between exposure and NDD onset, the role of environmental factors and their function in NDDs is not well known [22]. 

**Figure 1:**
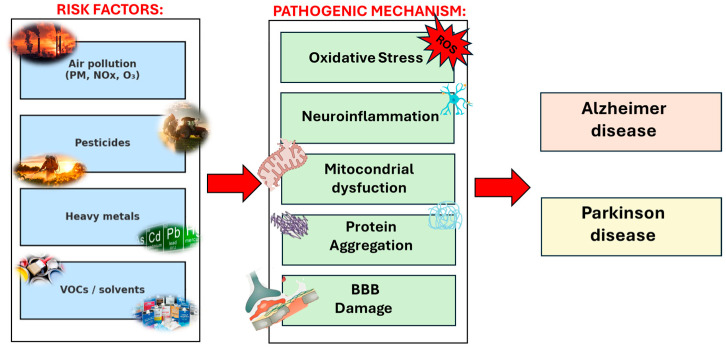
Pathogenetic mechanisms linking environmental exposures (PM, pesticides, heavy metals, VOCs) to neurodegenerative diseases such as AD and PD.

An increasing number of epidemiological and experimental studies suggest that environmental exposures to air pollution, including gaseous and particle pollutants, pesticides, heavy metals, and solvents, contribute to the pathogenesis of neurodegenerative diseases (NDDs), especially Parkinson’s disease (PD) and Alzheimer’s disease (AD) [23,24,25,26,27,28,29,30,31,32]. More specifically, overall, epidemiological, in vivo, and in vitro studies converge on the notion that environmental contaminants can cross the blood–brain barrier and induce oxidative stress [33,34,35,36], neuroinflammation [37,38,39], mitochondrial disfunction [40,41,42], and protein aggregation [43,44,45] (Figure 1). These processes represent shared pathogenic mechanisms contributing to the initiation and progression of NDDs such as PD and AD.

### 1.3. State of the Art on the Main Environmental Risk Factors

Although genetic determinants of neurodegenerative diseases (NDDs) have been extensively studied, the influence of environmental risk factors is only recently gaining comparable attention [33,34,35,36]. This section provides an overview of the current state of research addressing the association between environmental exposure and NDD onset, with particular attention paid to Parkinson’s disease (PD) and Alzheimer’s disease (AD). Hu et al., 2019, conducted a systematic review using meta-analysis data and estimated an increased risk of PD onset with a 10 ppb increase in nitrogen oxides (NOx) exposure, 1 ppm increment in carbon monoxide (CO) exposure, and 1 ppb increment in nitrogen dioxide (NO_2_) and ozone (O_3_) exposure, respectively [23]. Moreover, agricultural activity has been found to be a risk factor of Parkinson’s disease, probably due to the use of pesticides, insecticides, and herbicides. Indeed, Ascherio et al., 2006, explored whether a significatively increased risk of PD is associated with pesticide exposure in a large cohort of US men and women, comprising more than 140,000 participants and 413 incident cases of PD [46]. They found that individuals exposed to pesticides (5.7%) showed an increased relative risk of about 1.7 (ranging from 1.2 to 2.3) that was similar in farmers and nonfarmers and unrelated to asbestos and solvent exposure, as well as other occupational exposures. Similar results were found through the meta-analysis of 19 peer-reviewed studies performed by Priyadarshi et al., 2000 [47]. More specifically, the authors found an increased risk of PD incidence of about 1.94 (ranging from 1.49 to 2.53) in subjects exposed to pesticides, highlighting an association with the exposure duration. Further confirmation of the link between environmental exposure and the onset of Parkinson’s disease (PD) was provided by Corrigan et al. 2000, who reported high levels of organochlorine insecticides in the substantia nigra of deceased PD patients [48]. In this regard, in vitro experimental studies have shown that several compounds commonly included in pesticide formulations revealed to be involved in oxidative stress, interference with dopamine transporters, and mitochondrial dysfunction, ultimately leading to dopaminergic degeneration in the substantia nigra and motor abnormalities [44,47,49,50]. 

Another risk factor that has been found to be associated with the onset of PD is occupational exposure, particularly exposure to hydrocarbon solvent substances. These compounds have been found to involve the etiopathogenesis of PD because they probably have a key role in mitochondrial impairment, intraneuronal aggregation of phosphorylated α-synuclein protein and specific regional degeneration of nigrostriatal dopaminergic neurons [51]. Among hydrocarbon solvent substances, trichloroethylene (TCE) is a commonly used industrial solvent. Owing to its low molecular weight and lipophilicity it is rapidly and extensively absorbed following all routes of exposure and subsequently distributed throughout body tissues via systemic circulation. In particular, trichloroethylene can cross the blood–brain barrier causing neurological impairments (NIs) including neuropathies, cognitive, motor and sensory impairments and neurodegenerative diseases (NDGs) including Alzheimer′s and Parkinson’s disease [52].

However, the in vivo and animal studies conducted and published have shown to date limited and controversial results. Guehl et al., 1999 found that mice treated with TCE showed up to 50% dopaminergic DA neuronal death [53]. However, Liu et al., recently demonstrated that the progressive and selective loss of nigrostriatal dopaminergic neurons linked to chronic TCE administration to rodents is due to a potential neurotoxin, namely 1-trichloromethyl-1,2,3,4-tetrahydro-β-carboline (TaClo), that is endogenously built up from TCE rather than TCE alone [51,54]. Another VOC that has been found to be involved in the etiopathogenesis of PD is methanol. Methanol is a highly toxic alcohol included in gasoline as an additive, windshield washeries, antifreeze, paint strippers, and aerosol spray paints. It is known that the toxicity of methanol is due to its metabolites. Methanol is firstly metabolized to formaldehyde by alcohol dehydrogenase in the liver, and then to formic acid [55]. Several studies, in fact, have demonstrated the link between these metabolic products, especially formic acid, and neurological deficits characterized by lesions on the basal ganglia and different areas of white matter [55,56]. 

Regarding Alzheimer’s disease (AD), the 2020 Lancet Commission on Dementia Prevention identified ambient air pollution as one of three newly recognized risk factors for dementia, citing emerging evidence linking air pollution to increased Alzheimer’s disease (AD) susceptibility [57]. Studies in the literature, in fact, showed the relation between the pathology’s onset and progression and the exposure to environmental air pollutants such as particulate and nanoparticles, heavy metals, polychlorinated biphenyls (PCBs), polychlorinated dibenzo-p-dioxins (PCDDs), polychlorinated benzofurans (PCDFs),polycyclic aromatic hydrocarbons (PAHs), volatile organic compounds, and pesticides [22,58,59,60,61,62,63]. Crous-Bou et al., 2020 reported that exposure to air pollutants such as nitrogen dioxide (NO_2_), fine particulate matter (PM_2.5_), and coarse particulate matter (PM_10_) affects the neuroanatomical features and brain structure of healthy individuals [64]. Similarly, Calderon-Garciduenas (2019) revealed that subjects living in urbanized settlements and exposed to nano-sized particulate matter related to vehicular traffic (both combustion- and friction-derived particles) exhibited neuropathological features characteristic of AD [65]. More specifically, large-scale epidemiological studies have demonstrated a correlation between long-term PM_2.5_ exposure and cognitive decline and AD diagnosis [66,67]. A meta-analysis performed on 49 studies by Gong et al., 2023 showed a significant positive association between long-term PM2.5 and PM10 exposure and AD and PD with a pooled OR of 1.30 (95% CI: 1.14, 1.47, I^2^ = 99.3%) and 1.17 (95% CI: 1.00, 1.33, I^2^ = 91.8%), respectively [68]. Moreover, the authors found a pooled effect of Alzheimer’s disease with each 10 μg/m^3^ increase in exposure to PM2.5 (OR: 1.65–95% CI: 1.37, 1.94, I^2^ = 98.2%).

Similarly to findings about PD, environmental exposures to pesticides and heavy metals have garnered attention for their potential role in AD pathogenesis. Even if there is no significant association between plasma levels of PCBs or organochlorine pesticides (OCPs) and incident dementia or AD, elevated concentrations of PCBs and OCPs such as dichlorodiphenyltrichloroethane (DDT) and its metabolite dichlorodiphenyldichloroethylene (p,p′-DDE) were found in the blood of patients showing reduced cognitive performance [61,69,70]. Regarding exposure to heavy metals, multiple studies have consistently supported a link between exposure to elevated levels of aluminum, cadmium, chromium, copper, and iron and increased AD risk, as well as with cerebrospinal fluid (CSF) biomarkers such as p-tau and other pathological indicators of AD [71,72]. Moreover, animal studies performed on mice brains exposed to nickel demonstrated an increase in amyloid-β42 and amyloid-β40, which are associated with AD [39]. Finally, both in vitro and in vivo experimental studies have also demonstrated the relation between VOC exposure and AD. More specifically, studies involving laboratory animals exposed to benzene, toluene, ketone, alcohol, phenols, and other solvents showed biochemical and morphological changes in the neurons [73]. In addition, high levels of VOCs were detected in biological matrices (such as blood, breath and feces) from AD patients compared to levels from healthy individuals [74,75,76,77]. More recently, Xiong et al. (2025) used wearable air quality sensors to monitor the PM and VOC exposure of 59 elderly subjects, showing a significant correlation between these pollutant levels and AD biomarkers such as tau and amyloid positron emission tomography (PET) signals [78].

It should be emphasized that, in the case of AD, an association has also been reported between exposure to persistent air pollutants (POPs) and disease onset. Matés et al., 2010, indeed, reported that exposure to dioxins, emitted from combustion processes and certain industrial activities, affects the CNS Ca^2+^ homeostasis process and increases tau phosphorylation in neuronal cells through overexpression of P-glycogen synthase kinase-3β, consequently inducing oxidative stress [79]. Likewise, in studies on adult mice, it has been found that brominated flame retardants (BFRs) like polybrominated diphenyl ethers (PBDEs) commonly used in building materials, electrical appliances, and textiles, cause Ca^2+^ ATPase inhibition and apoptosis, which is the most common key factor observed in AD [22,80,81]. 

In summary, considering the experimental and epidemiological evidence as well as meta-analysis studies available to date, it is reasonable to hypothesize that exposure to air pollution may lead to NDDs onset due to cerebrovascular damage, neuroinflammation, Aβ peptide accumulation and oxidative stress [37,38,39]. Air pollutants including particulate matter (PM) and gaseous pollutants are able to reach the CNS via hematogenous routes or through direct inhalation via the olfactory epithelium. These pollutants can exert neurotoxic effects by compromising the integrity of the blood–brain barrier (BBB), thereby facilitating the entry and bioaccumulation of additional toxicants within the brain parenchyma [82]. This process activates resident immune cells, particularly microglia, and initiates neuroinflammatory cascades that may potentiate aging-related signaling pathways and promote progressive neurodegeneration [70]. Once air pollutants cross the BBB, they can act as toxic agents by a mechanism mainly linked to oxidative stress, which is the typical characteristic of neurodegenerative disease [83]. ROS formation or the alteration of the antioxidant’s enzymes contributes to the formation of protein aggregates such as Aβ, α-syn, and Tau proteins and leads to neuronal loss in specific regions of the brain such as the hippocampus and the cerebral cortex. In fact, it has been documented that environmental contaminants could enhance amyloid-β (Aβ) peptides along with tau phosphorylation, resulting in the initiation of senile plaques and neurofibrillary tangles, which results in the death of neurons [22,84]. 

## 2. VOCs in Biological Matrices as Potential Biomarkers of Neurological Diseases

### 2.1. A Preliminary Overview

Endogenous VOCs produced in the human body as a result of cellular metabolic processes have proven to be meaningful for clinical research. Volatile molecules as the output of metabolic processes can diffuse across cell membranes all over the body and are transported via the bloodstream from the site of origin to other parts of the body to be finally excreted through body fluids. Moreover, upon reaching the lungs, following the gas exchange mechanisms occurring at the alveolar level, they can easily pass into the exhaled air and are emitted by the human subject in the form of breath. Based on these assumptions, VOC determination in all body fluids may be useful for the early detection of several pathological conditions, including NDDs.

From a general point of view, an altered metabolism at the cellular level may lead to the generation of new metabolites and/or lead to changes in the associated physiological levels [85]. Among the factors related to the pathogenesis of NDDs, two interrelated processes are worthy to be mentioned, namely the mitochondrial dysfunction caused by pathogenic mutant proteins and the promoted production of reactive oxygen species (ROS), which is responsible for cellular damage, the generation of VOCs, and alterations in physiological concentrations. Lipid peroxidation, moreover, represents a key metabolic pathway involving free radicals and is responsible for the production of a pattern of compounds, the most important being aldehydes that may serve as biomarkers of different pathological conditions. One of these, hexanal, is currently a recognized biomarker of cellular oxidative stress [86]. As neurons are particularly rich in lipids, it has been speculated that oxidative stress may play a role in the pathogenesis of NNDs; however, to date, a strict correlation with the onset of NDDs has not been proven, and further research is needed to fill this important gap [85]. 

**Figure 2:**
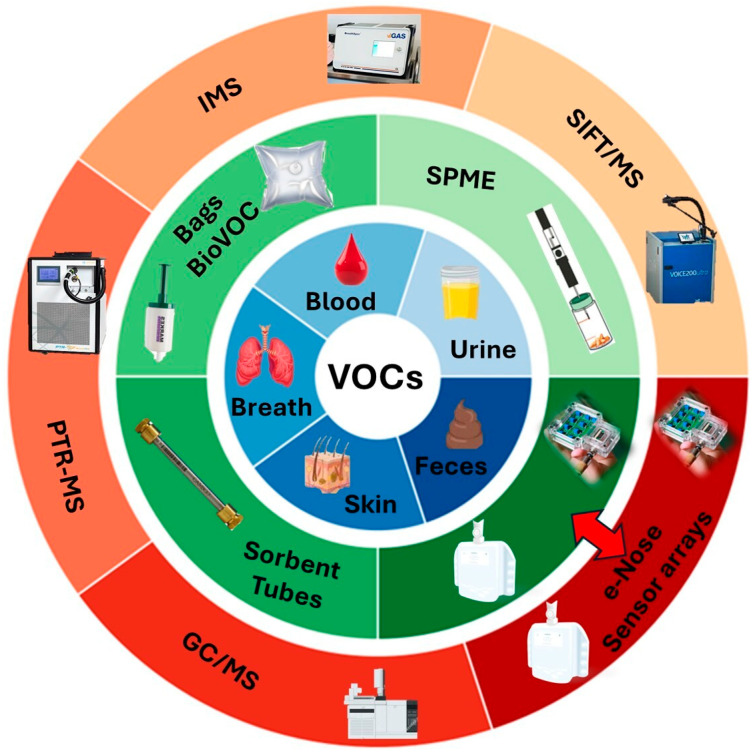
Overview of the methodological approaches for VOCs sampling and analysis in the volatome of different human biological matrices.

Although the identification of a disease-related VOC pattern in biological matrices may provide useful information on the health status of an individual, the clinical validity of VOCs as biomarkers is still challenging due to the complexity of the investigated human matrices, in terms of both sampling and analysis [87]. In this context, currently available methodologies in analytical chemistry along with recent advances in this field are undoubtedly strategic in the identification of the chemical profiling, e.g., fingerprint of complex matrices, including biological matrices [88]. Research on VOCs as biomarkers requires analytical techniques characterized by high levels of practicability, sensitivity, and selectivity. In the literature, a wide range of methodological approaches and analytical techniques have been applied for the collection and chemical characterization of human biological matrices, and, more specifically, for the identification of VOCs as biomarkers of neurodegenerative diseases. From a general perspective, the choice of the more suitable sampling procedure for the collection and extraction of VOCs from the matrix strictly depends on the nature of the biological matrix to be investigated (Figure 2). Furthermore, the selection of the analytical technique is mainly related to the purpose of the investigation (if targeted or untargeted analysis, if on-line or off-line analysis) and to the sensitivity required. Several analytical techniques are applied for VOCs chemical characterization in biological matrices: chromatographic techniques such as Gas Chromatography coupled with Mass Spectrometry (GC/MS), preceded by Thermal Desorption (TD-GC/MS) in the case of VOC collection onto adsorbent cartridges or Solid-Phase Micro Extraction (SPME) in case of use of coated fibers, and Gas Chromatography-Ion Mobility Spectrometry (GC-IMS).

In addition, over the years, direct mass spectrometry techniques such as Proton Transfer Reaction-Mass Spectrometry (PTR-MS), Secondary Electrospray Ionization Mass Spectrometry (SESI-MS) and Selected Flow Tube Ion-Mass Spectrometry (SIFT-MS) have exhibited high sensitivity and high performances for the real-time monitoring of VOC concentrations in several matrices [89,90]. Taking into account that high-performance analytical techniques are expensive, time-consuming, and require the specialized personnel, contextually to their application we are also witnessing over the last decade to the development and validation of innovative sensor technologies addressed to both chemical classes detection, e.g., e-Nose, and targeted gas detection, e.g., acetone, methanol, and formaldehyde [91,92,93,94]. These innovative technologies have been, to date, specifically designed and validated for the detection and high-temporal resolution monitoring of organic and inorganic gases of interest, principally in the exhaled breath or in the headspace of other biological matrices; therefore, they are beneficial for diagnostic purposes in point-of-care applications [93]. 

Finally, advanced statistical treatments of collected data represent a necessary step for extrapolating useful chemical information for classification and prediction. Recently, artificial intelligence (AI)-powered models are proving more effective in the detection of several diseases, as well as in the prediction of disease severity, in comparison with other statistical approaches. Methods such as machine learning (ML) and deep learning (DL) are increasingly emerging in recent years as strategic statistical tools in the early detection of human diseases, including NDDs, in the discovery of biomarkers, and personalized therapeutic treatment development [95]. However, in the NDD research field, the ability of AI-powered models to extract critical information from raw data has shown to be maximized when large-volume data are treated, e.g., genetic data, imaging data, clinical data, as it will be highlighted in the following sections when the outcomes of the existing studies on VOC determination for NDD detection and classification are reported and discussed.

Regarding the human biological matrices, exhaled breath, sebum and feces are the most studied sources in the human body for the diagnosis of NDDs through the analysis of volatome. The application of the aforementioned sampling methodologies and analytical techniques for VOCs characterization in the biological samples has allowed over the years the identification of a pattern of potential biomarkers for the early detection of several types of NDDs, e.g., AD, PD, MS, and ALS. In the following sections, a review of the scientific literature on the topic is described and discussed, highlighting the most significant outcomes obtained by the application of analytical methodologies on human breath, sebum, and feces (Section 2.1, Section 2.2, Section 2.3 and Section 2.4).

### 2.2. VOCs in Human Breath as Biomarkers of NDDs

Among the potentially investigable human body fluids, exhaled breath is the preferred matrix for the analysis of the volatile organic fraction (VOC) exhibiting several advantages and guaranteeing limitless availability in terms of size and sampling frequency.

It has been exhaustively speculated that altered metabolic pathways at the cellular level result in a distinctive VOC signature that can be detected in human breath, namely volatome, containing a plethora of volatile organic and inorganic compounds [96,97]. Exhaled breath is a complex biological matrix mainly composed of inorganic compounds such as nitrogen (N_2_), oxygen (O_2_), carbon dioxide (CO_2_), carbon monoxide (CO), nitric oxide (NO), water vapor (H_2_O) and, to a smaller extent, by a mixture of VOCs. Up to 3000 VOCs have, to date, been detected in human breath belonging to several chemical classes such as saturated and unsaturated hydrocarbons (alkanes, alkenes, branched-chain alkanes, and alkenes), aromatic hydrocarbons, alcohols, aldehydes and branched-chain aldehydes, ketones, carboxylic acids [98,99,100,101]. 

VOCs in human exhaled breath may be of both exogenous and endogenous origin. Exogenous VOCs are airborne organic compounds present in the external environment that may be inhaled by the human subjects during each individual inspiratory act and may follow two distinct principal routes: metabolism and excretion, or immediate re-emission into the environment through exhalation [98]. Endogenous VOCs are the products of complex metabolic processes occurring in different parts of the body. Although not directly produced in the lungs, they easily reach the airways via the blood stream, and through diffusive processes at the level of the alveolar capillary membrane they are expelled through exhaled breath. It is therefore reasonable to assume that breath VOC composition, i.e., the breathprint of each individual in each expiratory act, may provide a ‘snapshot’ of the cellular physiological state of the human subject and thus of the overall health condition. The current knowledge on endogenous VOCs excreted by the human body undoubtedly represented a starting point for the research on NNDs [99]. Similarly to the other human diseases investigated through breath analysis, the differentiation between healthy subjects and individuals affected by NNDs has to be based on the identification of a pattern of VOCs rather than on single compounds able to provide insight into specific pathological changes in neuronal cells.

Extensive scientific research in the field has been recently addressed to fill the knowledge gaps on metabolic pathways and on cellular mechanisms underlying the production of metabolites. For most of the VOCs that are detectable in human breath, their metabolic origin remains unclear and needs to be elucidated. Furthermore, being a complex matrix, sampling and analysis analytical steps are of paramount importance and are still under standardization at an international level [102,103]. The lack of a standardized methodological approach for exhaled breath sampling is undoubtedly recognized by the scientific community as the main factor that can lead to great variability in the analytical results. Several key aspects regarding breath sampling must be taken into account and have been faced by experts in the field over the years: (a) potential contamination of the sample due to exogenous factors (environmental contamination); (b) potential contamination due to the sample handling through several analytical steps; (c) the breath portion collected to maximize the intrinsic information in terms of metabolites [104]. The impact of different sampling techniques on the scientific soundness of breath analysis outcomes and their comparability worldwide were exhaustively assessed [105]. The sampling procedure generally relies on asking individuals to take a deep breath and to exhale until the completion of exhalation inside polymeric bags of different chemical compositions or other suitable containers (stainless steel canisters, glass or rigid polymeric containers), followed by direct analysis or transfer onto multi-bed adsorbent cartridges suitable for the analysis [106]. Over the last decade, automatic samplers that are able to collect the exhaled breath directly onto adsorbent cartridges have been designed and developed in order to optimize the entire sampling procedure. The use of such devices, indeed, has undoubtedly improved the sampling procedure by minimizing the breath sample contamination due to VOCs emission from container materials, e.g., polymeric materials, and to the sample handling over multiple analytical steps, e.g., sample transfer from the container to the analytical support. Finally, VOC extraction from the breath sample can also be performed exposing a coated fiber directly to the gaseous sample over a defined period of time. To overcome specific intrinsic limitations related to breath sampling and, more specifically, to collect a sample as representative as possible of the gas exchange at the capillary alveolar membrane, it has been suggested to preferentially sample the alveolar or ‘end-tidal’ fraction of the exhaled breath (generally the last 300 mL of the exhaled breath) instead of performing the collection of the entire exhalation flow—the so-called ‘mixed-breath’. This choice has been exhaustively justified, taking into account that endogenous VOCs indicative of cellular metabolism and potential alterations are concentrated in the end-tidal fraction of the exhaled breath coming from the inner airways and enriched with VOCs directly from the blood stream. On the contrary, the mixed breath also contains the anatomical ‘dead space air’ which is 150 mL of air coming from the upper airways (nose, mouth and trachea); this air lacks chemical information in terms of endogenous VOCs and determines, at the same time, dilution of the alveolar fraction [105,107]. Alveolar breath sampling can be carried out by “timed sampling”, performed automatically, or in “controlled” mode by measuring the CO_2_ concentration exhaled during a single exhalation or for several respiratory cycles.

Furthermore, VOCs in exhaled breath are generally present at trace levels, at ppb up to ppm levels [98,99]. As a result, a pre-concentration step may be useful to facilitate analytical detection. Several studies based on breath analysis relied on the preliminary concentration of breath samples, e.g., SPME [108]. 

With regard to the application of breath analysis in terms of VOCs characterization for research on NDDs, herein the main outcomes of the existing studies in the literature are presented and discussed. The first study was carried out by Wang et al., 2016, with the specific purpose to identify potential biomarkers for ALS able to discriminate between patients affected by ALS and patients suffering of Cervical Spondylotic Myelopathy (CSM), an ASL-mimicking disease exhibiting similar symptoms [94]. From a methodological point of view, prior to the GC-MS analysis the collected breath samples were subjected to a pre-concentration step applying SPME, e.g., exposing a 75 μm thick carboxen/polydimethylsiloxane polymeric fiber to the gaseous sample. The collected data (chromatographic peak areas) were normalized and processed applying multivariate statistical analysis methods, namely Principal Component Analysis (PCA) and Orthogonal Partial Least-Squares Discriminant Analysis (OPLSDA). Potential metabolic biomarkers were selected based on variable importance in the projection (VIP) values calculated from the PLS-DA supervised model and the area under the ROC curve (AUC) values were calculated to estimate the diagnostic accuracy of the model in distinguishing ALS from CSM. Statistically significant differences in terms of breath composition were found between the population groups of ALS and CSM and a specific pattern consisting of 4 VOCs, e.g., carbamic acid, monoammonium salt, 1-alanine ethylamide, N,N-dimethyl-guanidine and (p-hydroxyphenyl)-phosphonic acid was identified as a potential biomarker pattern of ALS, as detected at decreased level in neurological patients. The statistical analysis revealed that the AUC of the ROC curve for the biomarkers pattern (combined index) was 0.9643 (95% CI 0.9023–0.1000) with an accuracy of 92.68%, sensitivity and specificity equal to 0.929 and 0.923, respectively. A similar methodological approach (SPME-GC/MS) was applied by Lau et al., 2017, for the chemical characterization of breath samples collected from patients with Alzheimer’s disease (AD, n. 20) and Parkinson’s disease (PD, n. 20) and the identification of a VOCs pattern for the discrimination of the single neurological pathology with respect to healthy controls (n. 20) [91]. Similarly to Wang et al., 2016, the applied methodological approach for VOCs collection relied on the exposure of a 65 μm thick scoated fiber (polydimethylsiloxane/divinylbenzene) to the exhaled breath of single individuals collected inside 3 L Tedlar bags. One-way analysis of variance (ANOVA) was applied to chromatographic peak areas to highlight potential statistical differences between the AD, PD and control groups. The study revealed that selected VOCs such as 1-phenantherol, ethyl 3-cyano-2,3-bis (2,5,-dimethyl-3-thienyl)-acrylate were more abundant in the breath of patients affected by AD compared to the breath of patients with a diagnosis of PD and healthy controls. Moreover, the outcomes of the study allowed to speculate that ethyl-3-cyano-2,3-bis(2,5,-dimethyl-3-thienel)-acrylate and 1-phenanthherol have a strong association with disease progression, the latter likely related to endocrine disruption or acetylcholinesterase accumulation in synapses.

Another study based on GC-MS application was carried out by Akman et al., in 2018 and was aimed at investigating the potential correlation between neurological diseases and hexanal in the exhaled breath, recognized in the literature as biomarkers of lipid peroxidation of both ω-6 and ω-7 FAs [109,110,111]. In this study, breath samples was collected by individuals affected by multiple sclerosis (MS, n. 9) and Parkinson (PD, n. 8) as well as healthy controls by mean of the Bio-VOC device (Markes International), a system allowing to collect the exhaled breath directly onto suitable adsorbent tubes (Tenax-TA) prior to GC/MS analysis, minimizing in this way the potential sample contamination over the collection step.

The statistical treatment of hexanal abundances in the exhaled breath of the enrolled individuals was therefore performed via one-way ANOVA. According to the obtained results, a statistically significant difference in terms of hexanal levels was found between MS patients and healthy controls groups (*p*-value, 0.048). The higher hexanal levels detected in patients affected by MS may be reasonably associated with a promoted lipid peroxidation. Although lipid peroxidation has been documented as playing a key role in many neurological diseases due to the lipid-rich membranes of nerve cells, in this study, a correlation between the hexanal levels in the breath and Parkinson’s disease was not clearly found.

In another pilot study, Tiele et al. in 2020 explored the potentialities of breath analysis as a non-invasive diagnostic tool to discriminate between patients affected by Mild Cognitive Impairment (MCI) and healthy controls and between individuals with a diagnosis of Alzheimer’s disease (AD) and healthy controls, as well as to determine whether there were statistically significant differences in terms of VOCs composition between the exhaled breath of MCI and AD subject groups [77]. An overall number of 100 subjects were recruited in the study, divided as follows with age and genders approximately balanced: n. 50 healthy controls, n. 25 affected by AD, and n. 25 affected by MCI. The VOC characterization of the collected breath samples was performed, applying the GC-IMS technique as the analytical platform. The GC-IMS technique can measure compounds in the low parts-per-billion (ppb) range and delivers 10 min resolution data. The possibility to perform on-line measurements and to obtain high temporal resolution data represents one advantage of GC-IMS technique compared to the standard GC-MS, in the view of developing a diagnostic technique able to provide a rapid response for screening purposes. The end-tidal fraction of the exhaled breath (the last portion, approximately 350 mL) was directly sampled by each enrolled subject, by asking subjects to breath normally through a disposable mouthpiece connected at the instrument inlet (BreathSpec, G.A.S., Dortmund, Germany), and then analyzed.

After a pre-processing step to reduce the dimensionality of the acquired IMS data, a supervised feature selection was undertaken. Following the application of Wilcoxon rank sum test, feature exhibiting statistically significant differences among groups were selected to construct models based on five different classifiers, e.g., Support Vector Machine (SVM), sparse logistic regression, Gaussian process, Artificial Neural Network (ANN), and Random Forest (RF). This study highlighted that a selected VOCs pattern consisting of acetone, 2-propanol and 2-butanone may be considered predictive of Alzheimer’s disease and the model based on the aforementioned features was useful for the discrimination between AD and HC groups with the following parameters: AUC ± 95% equal to 0.83 (0.72–0.94), sensitivity of 0.60 (0.39–0.79), and specificity of 0.96 (0.80–1.00). The observed changes in the acetone levels in AD patients with respect to the physiological levels may be related to the region-specific declines in brain glucose metabolism occurring at the early stage of the disease. Acetone along with other ketone bodies such as acetoacetate, b-hydroxybutyrate is specifically produced from fat deposits to supplement glucose in patients affected by AD, often suffering loss of appetite. Similarly, changes in the breath levels of other common respiratory markers, such as 2-propanol, hexanal, heptanal, and 1-butanol, were revealed to be significant in the discrimination between AD patients and MCI patients and in the separation of HC and MCI. In the case of AD patients vs. MCI patients, the model performances in the discrimination between groups were as follows: AUC value 0.70 (0.55–0.85), sensitivity equal to 0.60 (0.39–0.79), and specificity equal to 0.84 (0.64–0.95). In the case of the HC and MCI patient groups, instead, the model performances parameters were AUC value 0.77 (0.64–0.90), sensitivity equal to 0.68 (0.46–0.85), and specificity equal to 0.80 (0.59–0.93).

In the cross-sectional study carried out by Tisch et al., 2013, the effectiveness of breath analysis in the early detection of neurodegenerative diseases, more specifically AD and PD, was further assessed [75]. A study population including 15 AD patients, 30 PD patients, and 12 HC (approximately aged and gender-matched) was investigated by coupling SPME-GS-MS analysis of the collected breath samples with nanomaterial-based sensor measurements. More specifically, an array of 20 organically functionalized nanomaterial-based sensors was validated in the study through comparison with data deriving from GC-MS chemical characterization. Therefore, the study represents the first example of an analytical–sensoristic integrated approach for the discrimination and classification of NDDs

The alveolar fraction of the exhaled breath was collected inside multi-layer sampling bags (polymeric and aluminum layer-Mylar bags), avoiding the simultaneous collection of dead space air. Computerized analysis of the overall sensor-array responses and selection of the most suitable sensing features using Discriminant Factor Analysis (DFA) provided ‘breath signatures’ and allowed to develop classification models able to clearly distinguish among the study populations. More specifically, patients affected by AD from healthy controls were discriminated with a classification accuracy equal to 85%, sensitivity equal to 93%, and specificity of 75%. A second DFA model distinguished well between the PD patients and the HC, exhibiting an accuracy of 79%, and sensitivity and specificity equal to 70% and 100%, respectively. Similar promising outcomes were obtained in the classification process between the individuals affected by the two neurodegenerative diseases. In detail, the sensitivity, specificity, and accuracy exhibited by a third model for the discrimination between AD and PD patients were estimated as 80%, 87%, and 84%, respectively. The chemical characterization of the collected breath samples supported the sensors outcomes highlighting the statistically significant differences among the study groups in terms of average abundance of a specific VOCs pattern. The study revealed that the average abundance of styrene, 2,3,6,7-tetramethyloctane, ethylbenzene and 1-methyl-3-(1-methylethyl)benzene were higher in the breath of PD patients with respect to the healthy controls, while a specific VOCs pattern consisting of styrene, 1-methyl-3-(1-methylethyl)benzene, 4-methyloctane, 2,6,10-trimethyldodecane, 3,7-dimethyldecane, butylated hydroxytoluene and 2,4-dimethyl-1-heptene was recognized as tentative biomarkers of AD. The outcomes reported in the study by Tisch et al., 2013, although not overlaid with those mentioned in previous studies, may find logical explanations in the neurodegenerative process underlying the two investigated diseases [75]. The increased abundance of alkanes and methylated alkanes found in breath samples of both AD and PD patients can be associated with promoted oxidative stress and lipid peroxidation involving cell phospholipids and resulting in cell-wall breakdown as well as with dopaminergic neuron death with specific regard to PD [112]. Multi-sensor-array systems are proving to be a promising candidate for the development of non-invasive biomedical devices, easy to use in ambulatory settings for screening activities prior to further and in-depth medical evaluations. Their versatility and ease of use by non-specialized personnel and patients themselves may, in the near future, promote breath analysis for the patient monitoring at home during the therapeutic treatment and/or in all those cases where recourse to specialized medical assistance is not always possible [113]. 

Another example of hybrid study based on the integration of analytical methodologies and sensoristic approaches is the study performed by Bach et al., in 2015 [114]. The authors employed a diagnostic tool based on the complementary application of an electronic nose device (Cyranose 320 eNose) and Ion Mobility Spectrometry (IMS) technique to detect breath pattern differences in the exhaled breath able to differentiate among patients affected by AD, PD and healthy controls. The overall study population recruited from two independent clinics consisting of n. 39 individuals suffering from AD, n. 16 patients with a diagnosis of PD and n. 35 healthy subjects was investigated. All study participants exhaled into a collection bag for e-Nose measurements and through a mouthpiece at the end of a Teflon tube directly connected to IMS instrumentation for on-line chemical analysis, therefore providing in both cases a mixed expiratory breath sample. In line with the results reported by Tisch et al., 2013 [75], the advanced statistical treatment with Linear Discriminant analysis (LDA) of collected e-nose data revealed significant differences between AD and PD (*p* < 0.0001), AD and HC (*p* < 0.0001) and PD and HC (*p* < 0.0001) with respect to distinctions between groups. The IMS analysis of breath samples, moreover, allowed us to tentatively identify five VOCs as potential biomarkers of both neurodegenerative disorders. In detail, the 2-ethyltoluene, 3-octanone and 2-methyfuran abundances in human breath of the investigated study population successfully differentiated AD patients from healthy individuals with a diagnostic accuracy equal to 94%. Finally, a decision-tree-based predictive model identified 1-butanol as the main biomarker of PD highlighting a differentiation of PD patients from the other two groups with 100% sensitivity and specificity.

Based on the description reported above, some considerations are worthy to be made. The analysis of the breath analysis-based studies herein explored clearly highlights a variability of the outcomes in terms of VOCs as potential biomarkers of NDDs. The observed variability of the data, in line with what has been already highlighted across studies focused on the application of breath analysis to other human diseases, may be related to several factors mainly attributable to the applied methodological approach. A standardized methodology at international level for breath sampling and analysis, indeed, capable of providing comparable and reliable results and minimizing inaccuracy in results interpretation is still lacking. Factors that can affect the qualitative and quantitative composition of the exhaled air in terms of VOCs include the portion of breath sampled (mixed expiratory breath versus end-tidal breath), the sample collection mode (single exhalation versus multiple exhalations) and the physiological parameters of the subject such as breathing rate, which may vary from subject to subject or for the same subject in separate exhalations and can determine a different rate of VOCs exhalation according to different exchange rates at the alveolar level [115]. As previously mentioned in the present section, the representativeness of the breath portion in relation to the gas exchange occurring at the level of the alveolar capillary membrane between the bloodstream and pulmonary air is still a subject of debate within the international scientific community [105,116,117]. Based on the current debate, the end-tidal fraction of the exhaled breath (generally, the last 300 mL of the exhaled breath) is considered more representative of the gaseous exchange with the blood in the alveoli, and therefore richer in chemical information related to cell metabolism and related disorders in individuals affected by different diseases [107]. Except for some limited overlaps between studies, e.g., styrene, 1-butanol and hexanal, in the light of the assumption above, the observed variability may be partially explained taking into account the different portion of breath collected and analyzed: mixed expiratory breath [91,114] or end-tidal breath [75,77,109]. Other factors affecting to a greater or lesser extent the variability of the results are the sample contamination by exogenous VOCs (present in the ambient air where the sampling procedure is performed) and the potential interference of VOCs produced by the microbiota in the oral cavity or in the gut [118]. These factors need to be considered in a critical evaluation of the reported outcomes. The different approaches applied across the studies to clear the inhaled air before breath collection might have been effective in minimizing the interference of exogenous VOCs, in comparison with other studies where this issue was not accurately addressed: the use, for instance, of automatic samplers operating a pulmonary wash-out [109] or repeated inhalations performed by the subject of standardized medicinal air [114], or through a mouthpiece containing a filter cartridge [75]. An alternative strategy to minimize the exogenous VOCs interference is to perform sampling sessions where the ambient air is simultaneously collected and analyzed. Therefore, VOC concentrations encountered in the ambient can be subtracted from the respective values in breath samples (when applicable) or appropriately included in the statistical treatment [103]. In addition, when the sampling is performed using suitable containers such as polymeric bags, the potential sample contamination due to VOCs emission from the polymeric materials may occur and should be adequately taken into account [91]. Significant variability among different study populations may also be reasonably attributed to confound factors such as diet, habits (smoking or alcohol consumption) or treatment-related VOCs [119,120]. 

Besides the methodological approach for breath sampling, discrepancies of the reported outcomes may also be related to the performances of the applied analytical techniques (VOCs collection/extraction efficiency, low detection limits, detection capability toward high volatility VOCs). Not less significant is the statistical approach chosen for data treatment. The successful extraction of the hidden information from the collected data, that means the identification of a VOCs metabolic pattern from the complex matrix of human breath, can also be trace back to the statistical data treatment applied on the dataset. It is widely accepted that the selected process for the extraction of valuable chemical information from complex metabolomics data addressed at the identification of potential biomarkers may significantly affect the outcomes and, as a consequence, contribute to the data variability. The selection of the proper statistical approach in relation to the characteristics of the collected dataset (sample size, being another important factor to be considered), indeed, seems to be a crucial step. ML approaches have been extensively used over the last years to process VOCs data deriving from breath investigations, showing effectiveness in processing large metabolomic datasets and in the development of mathematical models and learning algorithms for prediction, classification and decision making [113]. Among the discussed studies, it is possible to observe that ML-based classification approaches have been successfully applied in case of high-dimensionality data, e.g., GC-IMS data [77] and sensor-array outputs [75,114]. 

### 2.3. VOCs in Human Feces as Biomarkers of NDDs

An interesting application of the volatilome analysis of human feces is addressed to explore the relationship between the microbiome and NDDs development. Although it was already speculated in the past that the human microbiome plays a key role in the bidirectional communication between the GI tract and the CNS, the microbiota disruption has only recently been associated with the etiopathology of NDDs. Few recent studies therefore suggest that the dysbiosis of the gut microbiome may result in the release of a specific VOC pattern in the feces that is potentially indicative of NDDs [121,122]. Despite its great exploratory potential, the fecal volatilome appears to be less explored for diagnostic purposes than the exhaled volatilome, not only in relation to NDDs, but also in the broader context of human pathologies [123]. This can be mainly justified by the great complexity in data interpretation, which is more susceptible to specific confounding factors such as diet and gut microbiota activity. The standardization of both sampling and storage procedures, as highlighted for the exhaled breath, are of paramount importance to obtain reliable data and to allow comparability of results. Attention has been therefore paid across these studies to the optimization of the sampling procedure (sample collection and transport) and of the storage parameters (temperature and time), in order to avoid sample contamination and degradation [104]. The widely applied methodological approach for VOCs collection (sample headspace) and chemical characterization from fecal samples is HS-SPME coupled with GC-MS analysis. This is the same methodological approach applied in the study by Ubeda et al., 2023, where the potential association between fecal VOCs and the extent of cognitive impairment in patients affected by AD was tentatively explored [121]. Investigations were performed on a study population consisting of n. 10 healthy individuals and n. 12 AD patients, the latter differentiated in subgroups depending on the AD stage measured by Global Deterioration Scale (GDS): n. 4 at GDS-3, n.4 at GDS-4 and n. 4 at GDS-5. More specifically, VOCs and bacterial taxa were both characterized in the collected feces. Experimental data resulting from the chemical characterization of fecal volatilome for both the investigated population groups were statistically treated, firstly applying PCA to determine the most weighting variables in the differentiation between AD patients and HCs and successively by partial least squares discriminant analysis (PLS-DA) to further process the data and attribute to the detected VOCs the potential role of AD biomarkers, based on VIP score.

A pattern of 29 fecal VOCs belonging to seven different chemical classes (alcohols, esters, acids, terpenes, aldehydes, sulfur compounds, ketones) along with 13 bacterial genera revealed to be discriminant between the HCs and the AD patients, at all the stages of the cognitive impairment. With respect to the healthy controls, a specific pool of Short-Chain Fatty Acids (SCFAs), e.g., acetic, propanoic, butanoic, pentanoic, hexanoic, and heptanoic acids, yielded the highest amounts in the feces of AD patients. This evidence may be reasonably attributed to the activity of gut microbiota and, more specifically, to the fermentation of complex nutrients as the carbohydrates resulting in a promoted production of SCFAs [124,125], confirming recent studies that highlighted the association between an altered gut microbiota derived from the SCFAs and the pathogenesis of AD [126]. Moreover, trans-2-decenal was revealed to be positively correlated with AD patients, and is one of the most significant features in the differentiation between AD patients and HCs. A clear understanding of the biochemical pathway of trans-2-decenal is still needed, although the authors assume that the compound may be involved in protective mechanisms expressed by the gut microbiota against bacteria and fungi colonization.

In addition, a slight increase in 1-butanol abundance was also observed. Although in this case a metabolic pathway was not speculated, the detection of 1-butanol at higher levels in the volatilome of feces of AD patients compared with HCs represents an interesting matching with the outcomes of breath investigations documented in the study by Tiele et al., 2020 [77]. The study also highlighted a specific fecal volatile profile depending on the AD stage, allowing to distinguish the early stage without dementia (GDS-3) from the middle stages with dementia (GDS-4 and GDS-5). The early stage of AD was mainly characterized by a higher abundance of SCFAs with a low molecular weight, while at a more advanced stage in cognitive impairment, the volatile profile shifted towards a higher abundance of heptanoic and hexanoic acids. On the basis of the obtained outcomes, the authors also speculated a crosstalk between VOCs and microbiota in the progression of cognitive impairment.

The potential of the HS-GC/MS analytical technique in low-trace VOC detection was further highlighted in the study by De Pablo-Fernandez et al., 2022, who aimed to evaluate the fecal metabolome in PD patients [122]. According to specific clinical criteria, patients affected by PD and healthy individuals were recruited; one group of n. 35 PD patients and two control groups with no known neurological (n. 35) or gastrointestinal pathology (n. 15) were recruited. Statistical analysis of the collected data was performed by applying ANOVA. Based on the outcomes of the study, a VOC pattern was detected at an increased concentration in fecal samples of PD patients with respect to both control groups. More specifically, the abundances of 1,3-ditert-butylbenzene, butan-2-one, selected terpenes, e.g., γ-terpinene, α-pinene, o-cymene, R-D-limonene, 2-methylpropanal, 3-methylbutanal, 3-methylsulfanylpropanal and hexanal were higher in PD patients with respect to both control groups. In this study, among the aforementioned compounds, 1,3-ditert-butylbenzene was found to be strongly associated with the fecal metabolome of PD patients (found in 97% of PD patients and only in 11.4% of control subjects). No speculation about the origin and significance of 1,3-ditert-butylbenzene was made and is currently possible, based on the current level of knowledge. Therefore, further research is needed to potentially elucidate the role and significance of this compound. However, it has been already detected in the stool samples of subjects with coeliac disease [127]. 

### 2.4. VOCs in Human Sebum as Biomarkers of NDDs

Recently, the potential association between VOCs in human sebum and non-motor manifestations of PD such as skin disorders have also been explored [128,129,130,131,132]. Increased sebum secretion in PD patients was associated with increased production of yeasts and enzymes in the body, and hormone secretion can lead to seborrheic dermatitis. Seborrheic dermatitis is considered one of the non-motor symptoms of PD and has additional value for diagnosis. Moreover, pilot studies have recently highlighted that a characteristic smell from sebum of individuals affected by PD is detectable, thus foreshadowing new scenarios for the non-invasive diagnosis of the disease. VOC samples can be collected from a patient’s skin and sweat using several methodological approaches. The simplest consists of swabbing the patient’s skin surface with a contact medium (e.g., gauze, cotton ball) and analyzing the VOCs present in the gauze. An alternative approach to collect samples from patients’ skin and sweat is based on the use of a large-scale test chamber where under controlled conditions, the whole-body skin emissions from human subjects can be measured minimizing in this way the influence of environmental factors and guaranteeing the accuracy in VOCs identification. It is, however, important that patients follow strict bathing, dietary, exercise, and environmental protocols prior to skin and sweat collection. The sampling approach based on sebum collection on swabs was applied in the studies by Sinclair et al., 2021 [130] and Trivedi et al., 2019 [131], both addressed at the identification of a VOCs pattern in sebum as biomarkers potentially predictive of PD. In both studies, the chemical characterization in terms of VOCs composition of the collected sebum from population groups of PD patients and healthy controls by DHS-TD-GC/MS revealed the significance of a selected VOCs pattern in discrimination between PD patients and controls. In detail, the study by Sinclair et al., 2021 [130], confirmed the findings previously reported in the study by Trivedi et al., 2019, where the differential profile in the skin of individuals affected by PD was already highlighted based on a restricted pool of VOCs, namely perillic aldehyde, hippuric acid, eicosane and octadecanal [131]. A wider VOCs pattern potentially serving as a biomarker for PD was identified in the study by Tsuda et al., 2019 [132]. The modeling (partial least square-PLS, support vector machine-SVM and support vector regression-SVR analyses) of experimental data obtained by GC/MS analysis of skin wipes collected by patients affected by PD at different severity grade (n. 61 severe and mild PD patients) and by n. 61 healthy controls allowed to identify potential biomarkers and to differentiate them according to the disease severity. In detail, the candidate VOCs for predicting PD onset and severity are as follows: (a) nonanal, heptanal, 2-butenal, styrene, ethyl acetate, 6-methyl-5-Hepten-2-one, hexadecane, dodecane and acetone for severe PD; (b) decanal, hexanal, 2-ethylhexanol, ethylbenzene, xylenes, and N,N-dimethylacetamide for mild PD. Some of the candidate VOCs detected in the volatile fraction of skin sebum were also identified as significant in classification in other studies and mainly related to the promoted lipid peroxidation and oxidative stress at cellular level in PD patients, e.g., nonanal, heptanal, 2-butenal, dodecane. The candidate compounds for PD prediction and early diagnosis identified in this study may also be reasonably emitted from PD patients’ skin as a result of skin aberrations, e.g., abnormal skin permeability. A plausible hypothesis is that the identified VOCs are emitted by the skin of individuals affected by PD due to relevant changes in skin texture occurring contextually with the manifestation of the neurological impairment.

In 2022, Fu et al. proposed an advanced artificial intelligent olfactory (AIO) system based on the integration of fast GC with a surface acoustic wave (SAW) sensor to identify a specific VOCs fingerprint in the human sebum for the diagnosis of PD [129]. Similarly to investigations performed on breath samples by Tisch et al., 2013 [75], this is another example of integration of consolidated gas-chromatographic techniques with innovative sensor-based approaches. An overall number of 43 PD patients and 44 HCs were enrolled in the explorative study. Univariate and multivariate analysis was performed on a sub-dataset and ML-based classification approaches (k nearest neighbor-KNN, Ada Boost-AB, Naïve Bayes-NB, SVM and RF), were applied to provide biomarkers-based and odor profile-based models. The most significant features for discrimination between PD patients and HCs were octanal, hexyl acetate and perillic aldehyde. The octanal has been extensively associated in other studies to the promoted lipid peroxidation [111] and oxidative stress [133], highly evident in several NDDs, while for the other two identified compounds hypothesis on metabolic routes are lacking. The model developed on the three most significant features exhibited a classification accuracy equal to 70.8%. Promising outcomes were obtained for the model based on the odor profile showing a sensitivity of 91.7%, specificity of 91.6%, and AUC of 0.826. The AIO systema was therefore proposed as a fast and non-invasive tool to easily screen and monitor PD patients.

Despite the studies carried out so far, further research is required to elucidate the metabolic pathways disorders leading to the production of PD-related VOCs and, more specifically, the mechanisms underlying the emission through human skin.

VOCs detected in human breath, feces and sebum and proposed as potential biomarkers of NDDs over the existing studies in the literature (herein discussed) are summarized in a comprehensive list reported in Table 1. The chemical class of the identified VOCs and the speculated route of generation and/or role in cellular metabolism for the investigated NDDs are additionally reported.

### 2.5. Challenges and Controversies of VOCs Detection in Biological Matrices for Diagnostic Purposes: A Brief Discussion on Potentialities and Limitations

In the present section, the potentialities and limitations of human volatilome characterization in terms of VOCs determination, with the aim of identifying a reliable pattern of biomarkers of NDDs, are discussed. In addition to discovering more effective treatment protocols, a recognized priority in NDDs research field is nowadays the development and validation of a non-invasive population screening test aimed at early diagnosis of diseases as well as the implementation of prevention programs for individuals at potential risk based on genetic factors and/or specific predisposing factors. Of paramount importance is that screening tests are minimally invasive for the individuals in order to encourage social adherence to the prevention programs. This aspect is particularly important for NDDs, for which diagnosis is often delayed due to the difficulty of a proper interpretation of the early manifestation of the disease (difficulty in memorizing or spatial-temporal disorientation, movement impairment, etc.). Based on the current level of knowledge acquired over the last decades by the international scientific community, among the approaches herein explored, breath analysis exhibits a great potential to become a novel diagnostic approach for early diagnosis and personalized therapeutic management. Breath analysis potentialities, compared to conventional diagnostic approaches, are undoubtedly the non-invasiveness, the versatility and the cost-effectiveness. It was also revealed to be painless (if compared to blood sample collection, for instance), potentially straightforward to implement, compatible with the high frequency monitoring of the subject and, of no less importance, is well tolerated by vulnerable patients.

The non-invasiveness of the approach and the ease of performing can certainly increase the population’s adherence to screening campaigns, with particular reference to elderly individuals. Its accreditation as a screening method could have significant both clinical and socio-economic implications. The economic implications are referred to by reduced costs for the health system, while the social implications are in terms of prevention and general improvement in citizens’ quality of life. Despite its proven potential and undisputed strengths, breath analysis is not fully accredited as a diagnostic screening technique due to a still existing gap of knowledge about the biochemical processes underlying cellular metabolism, the several endogenous and exogenous factors affecting the breath VOC composition and the lack of standardized procedures for sampling and analysis able to guarantee the reliability and comparability of experimental results at all levels of the methodological approach. The application of different methodological approaches for the collection, pre-concentration and chemical characterization of VOCs of interest has not yet promoted the standardization of procedures, an essential step for the implementation of breath analysis in the healthcare system as a routine clinical diagnostic tool to support conventional diagnostic techniques. Moreover, one of the main limitations of breath studies focused on NDDs (also found across studies focusing on other diseases, both chronic and oncologic) is the sample size of the population groups. Most of the studies discussed in the present review are, indeed, pilot studies aimed at the exploration of the potentialities of breath analysis for biomarkers identification and are therefore characterized by a limited number of individuals for each NDDs category and of healthy controls. There is therefore the urgent need for validating breath analysis outcomes, e.g., VOCs pattern, for the early detection of NDDs within a larger sample size. Larger patient groups can be certainly considered more representative of a specific NDD-related population and the validation of breath approach within larger patient sample may provide more reliable scientific outcomes.

Similar considerations regarding the need for the enlargement of the study population are valid also for the other human sources investigated in relation to NDDs and discussed in the present review, e.g., feces and sebum. This objective can be successfully achieved through multicenter studies, representing one of the challenges to be pursued in the future.

Another important limitation that can be reasonably extended to all the biological matrices herein discussed is the limited knowledge about the etiology of NDDs and the mechanisms of neurodegeneration. Although mitochondrial dysfunction, oxidative stress, and lipid peroxidation have been proposed as the main metabolic pathways to explain the production and excretion of certain VOCs, there is still the need for further research in order to elucidate the origin and the role of extended VOC patterns. Another challenge, therefore, is to deepen the mechanisms of neurodegeneration as well as to provide new insights into the relationship between the gut microbiome and NDDs. By systematizing knowledge with a multidisciplinary approach and continuing research by exploring the volatilome of multiple biological matrices (including, for instance, urine that has been little or not at all explored in relation to NDDs), it will be possible to fill many presently existing knowledge gaps.

The long-term vision is to develop a multi-level prevention model. Starting with non-invasive, rapid and low-cost screening tests based on volatilome characterization of the exhaled breath or another easy-to-handle biological matrix, applying advanced analytical techniques and/or innovative sensor-based approaches, it is possible to proceed with further investigations using conventional diagnostic methods (more invasive for the patient, such as computed tomography or nuclear magnetic resonance imaging) for situations that are uncertain after the first-level screening.

## 3. Conclusions

Although several scientific studies in the literature demonstrated a significant positive correlation between human exposure to environmental air pollutants and neurodegenerative diseases onset and contributed to improving knowledge on disease etiopathogenesis and progression, there is still a lack of potential approaches for non-invasive early NDDs diagnosis. Moreover, taking into account environmental risk factors and, more specifically, air-pollution-induced neurotoxicity, more attention should be paid to human exposure to neurotoxic air pollutants. In this context, tailored strategies developed to improve air quality and reduce exposure to harmful pollutants could significantly reduce the incidence of certain neuro-degenerative diseases, and could advance the identification of biomarkers specific to NDDs and the development of non-invasive diagnostic approaches, allowing us to accurately monitor the progression and improve the management of NDDs worldwide.

## Figures and Tables

**Table 1:** VOCs in selected biological matrices, e.g., breath, feces and sebum, as potential biomarkers of NDDs. The chemical class and the speculated metabolic pathways are reported in brackets, when possible.

NDDs	VOCs in Main Biological Matrices as Potential Biomarkers of NDDs (Chemical Class—Metabolic Pathway)		
	Breath	Feces	Sebum
**AD**	**Styrene** (aromatic hydrocarbon—potentially linked to oxidative stress); **1-methyl-3-(1-methylethyl)benzene** (branched aromatic hydrocarbon—unknown); **4-methyloctane** (methylated alkane—oxidative stress and lipid peroxidation); **2,6,10-trimethyldodecane** (methylated alkane—oxidative stress and lipid peroxidation); **3,7-dimethyldecane** (methylated alkane—oxidative stress and lipid peroxidation); **butylated hydroxytoluene** (branched phenol—unknown) **2,4-dimethyl-1-heptene** (methylated alkene/potentially linked lipid peroxidation) [75]	**trans-2-decenal** (medium-chain fatty aldehyde—potentially associated with gut microbiota defense mechanisms); **acetic acid** (carboxylic acid—altered gut microbiota); **propanoic acid** (SCFAs—altered gut microbiota); **butanoic acid** (SCFAs—altered gut microbiota); **pentanoi acid** (SCFAs—altered gut microbiota); **hexanoic acid** (SCFAs—altered gut microbiota); **heptanoic acid** (SCFAs—altered gut microbiota); **1-butanol** (alcohol—unknown) **3-phenyl-propanol** (alcohol—unknown); **butyl 2-methylbutanoate** (ester—unknown); **propyl butanoate** (ester—unknown) [121]	
	**2-ethyltoluene** (aromatic hydrocarbon—unknown); **3-octanone** (ketone—unknown); **2-methylfuran** (heteroaromatic compound—unknown) [114]		
	**1-phenantherol** (polyaromatic hydrocarbon—possibly attributed to endocrine disruption or accumulation of acetylcholinesterase at the synapse); **ethyl 3-cyano-2,3-bis(2,5,-dimethyl-3-thienyl)-acrylate** (hydrocarbon—unknown) [91]		
	**acetone** (ketone—potentially linked to the decline in brain glucose metabolism); 2-propanol (alcohol); 2-butanone (ketone); **hexanal** (aldehyde—lipid peroxidation); **heptanal** (aldehyde—lipid peroxidation); **1-butanol** (alcohol—unknown) [77]		
**PD**	**1-butanol** (alcohol—unknown) [114]	**1,3-ditert-butylbenzene** (alkylated aromatic hydrocarbon—unknown); **γ-terpinene** (monoterpene—unknown); **α-pinene** (bicyclic terpene—unknown) **o-cymene** (monoterpene—unknown); **(R)-(d-limonene)** (monoterpene—unknown); **2-methylpropanal** (aldehyde—potentially related tolipid perxidation); **3-methylbutanal** (aldehyde—potentially related to lipid peroxidation); **hexanal** (aldehyde—lipid peroxidation); [122]	**octanal** (aldehyde—oxidative stress, lipid peroxidation) **hexyl acetate** (ester—unknown) **perillic aldehyde** (aldehyde—unknown) [129]
	**styrene** (aromatic hydrocarbon—potentially increased in PD patients breath due to oxidative stress); **2,3,6,7-tetramethyl-octane** (methylated alkane—oxidative stress and lipid perixidation; **ethylbenzene** (alkylated aromatic hydrocarbon—unknown); **1-methyl-3-(1-methylethyl)benzene** (alkylated aromatic hydrocarbon—unknown) [75]		**perillic aldehyde** (aldehyde—unknown) **hippuric acid** (carboxylic acid—associated with changes in skin microflora and physiology); **eicosane** (aliphatic hydrocarbon—associated with changes in skin microflora and physiology); **octadecanal** (long-chain aldehyde—increased and altered sebum secretion) [130,131]
			**2-butenal** (enal—lipid peroxidation); **hexanal** (aldehyde—lipid peroxidation); **heptanal** (aldehyde—lipid peroxidation); **nonanal** (aldehyde—lipid peroxidation); **decanal** (aldehyde—lipid peroxidation); **2-ethylhexanol** (alcohol—unknown); **styrene** (aromatic hydrocarbon—potentially linked to oxidative stress) **ethylbenzene** (aromatic hydrocarbon—unknown); **p-xylene** (aromatic hydrocarbon—unknown); **acetone** (ketone—potentially indicative of latent insulin resistance); **5-hepten-2-one** (ketone—unknown); **ethyl acetate** (ester—unknown); **hexadecane** (straight-chain alkane—unknown); **tridecane** (straight-chain alkane—unknown); **dodecane** (straight-chain alkane—potentially linked to lipid peroxidation); **6-methyl-o-xylene** (methylated aromatic hydrocarbon—unknown); **N,N-dimethyl acetamide** (amide—unknown) [132]
**MS**	**hexanal** (aldehyde—biomarker of lipid peroxidation) [109]		
**ALS**	**carbamic acid, monoammonium salt** (derivative of carbamic acid—decreased levels on ALS patients, involved in synthesis of diethyldithio-carbamic acid, the latter inhibitor of superoxide dismutase elevated in case of oxidative stress); **1-alanine ethylamide** (potentially involved in the synthesis of CDDO-ethylamides palying a role in the activation of the endogenous cytoprotective system); **guanidine, N,N-dimethyl** (decreased levels in ALS patients, inhibitor of NO synthesis); **phosphonic acid, p-hydroxyphenyl** (organophosphorus compound—decreased levels in ALS patients) [94]		

## Abbreviations

AD	Alzheimer’s Disease
ALS	Amyotrophic Lateral Sclerosis
AUC	Area Under the Curve
CI	Confidence Interval
CNS	Central Nervous System
CO	Carbon Monoxide
CSM	Cervical Spondylotic Myelopathy
CT	Computed Tomography
DHS-TD-GC/MS	Dynamic Headspace–Thermal Desorption GC-MS
DFA	Discriminant Factor Analysis
DL	Deep Learning
e-Nose	Electronic Nose
FRDA	Friedreich’s Ataxia
FTD	Frontotemporal Dementia
GC-MS/GC-IMS	Gas Chromatography–Mass Spectrometry/Ion Mobility Spectrometry
GDS	Global Deterioration Scale
HD	Huntington’s Disease
LBD	Lewy Body Dementia
LDA	Linear Discriminant Analysis
ML	Machine Learning
MRI	Magnetic Resonance Imaging
NDDs	Neurodegenerative Diseases
NOx	Nitrogen Oxides
NS	Nitrosative Stress
OCPs	Organochlorine Pesticides
OR	Odds Ratio
OS	Oxidative Stress
PAHs	Polycyclic Aromatic Hydrocarbons
PCBs	Polychlorinated Biphenyls
PD	Parkinson’s Disease
PET	Positron Emission Tomography
PLS-DA	Partial Least Squares Discriminant Analysis
PM2.5/PM10	Particulate Matter (2.5/10 μm)
POCT	Point-of-Care Testing
PTR-MS/SIFT-MS	Proton Transfer Reaction-MS/Selected Ion Flow Tube-MS
RF	Random Forest
ROS	Reactive Oxygen Species
SCFAs	Short-Chain Fatty Acids
SPME	Solid Phase Microextraction
TaClo	1-trichloromethyl-1,2,3,4-tetrahydro-β-carboline
SVM	Support Vector Machine
VIP	Variables with an Importance in Prediction
VOCs	Volatile Organic Compounds

## References

[1] Lamptey R.N.L., Chaulagain B., Trivedi R., Gothwal A., Layek B., Singh J. (2022). A review of the common neurodegenerative disorders: Current therapeutic approaches and the potential role of nanotherapeutics. Int. J. Mol. Sci..

[2] Liu H., Hu Y., Zhang Y., Zhang H., Gao S., Wang L., Wang T., Han Z., Sun B.L., Liu G. (2022). Mendelian randomization highlights significant difference and genetic heterogeneity in clinically diagnosed Alzheimer’s disease GWAS and self-report proxy phenotype GWAX. Alzheimers Res. Ther..

[3] Jain N., Chen-Plotkin A.S. (2018). Genetic modifiers in neurodegeneration. Curr. Genet. Med. Rep..

[4] Jain V., Baitharu I., Barhwal K., Prasad D., Singh S.B., Ilavazhagan G.J.C. (2012). Enriched environment prevents hypobaric hypoxia induced neurodegeneration and is independent of antioxidant signaling. Cell. Mol. Neurobiol..

[5] Emard J.F., Thouez J.P., Gauvreau D. (1995). Neurodegenerative diseases and risk factors: A literature review. Soc. Sci. Med..

[6] Ogonowski N.S., García-Marín L.M., Fernando A.S., Flores-Ocampo V., Rentería M.E. (2024). Impact of genetic predisposition to late-onset neurodegenerative diseases on early life outcomes and brain structure. Transl. Psychiatry.

[7] Bertram L., Tanzi R.E. (2005). The genetic epidemiology of neurodegenerative disease. J. Clin. Investig..

[8] Karran E., De Strooper B. (2022). The amyloid hypothesis in Alzheimer disease: New insights from new therapeutics. Nat. Rev. Drug Discov..

[9] Bisi N., Pinzi L., Rastelli G., Tonali N. (2024). Early diagnosis of neurodegenerative diseases: What has been undertaken to promote the transition from PET to fluorescence tracers. Molecules.

[10] Manera V., Rovini E., Wais P. (2023). Editorial: Early detection of neurodegenerative disorders using behavioral markers and new technologies: New methods and perspectives. Front. Aging Neurosci..

[11] Rekatsina M., Paladini A., Piroli A., Zis P., Pergolizzi J.V., Varrassi G. (2020). Pathophysiology and therapeutic perspectives of oxidative stress and neurodegenerative diseases: A narrative review. Adv. Ther..

[12] Islam M.T. (2017). Oxidative stress and mitochondrial dysfunction-linked neurodegenerative disorders. Neurol. Res..

[13] Santos R., Bulteau A.L., Gomes C.M. (2016). Neurodegeneration, neurogenesis, and oxidative stress. Oxid. Med. Cell Longev..

[14] Schulz J.B., Lindenau J., Seyfried J., Dichgans J. (2000). Glutathione, oxidative stress and neurodegeneration. Eur. J. Biochem..

[15] Fusco M., Skaper S., Coaccioli S., Paladini A., Varrassi G. (2017). Degenerative joint diseases and neuroinflammation. Pain Pract..

[16] Nathan C., Ding A. (2010). Nonresolving inflammation. Cell.

[17] Varrassi G., Fusco M., Skaper S.D., Battelli D., Zis P., Coaccioli S., Pace M.C., Paladini A. (2018). A pharmacological rationale to reduce the incidence of opioid induced tolerance and hyperalgesia: A review. Pain. Ther..

[18] Boncristiani C., Di Gilio A., De Castro F., Nardini A., Palmisani J., Martínez Vázquez R., de Gennaro G., Fanizzi F.P., Ciccarella G., Vergaro V. (2025). Recent advances in 3D models for multiparametric blood–brain barrier detection in microfluidic systems. J. Mater. Chem. B.

[19] GBD 2021 (2024). Global Burden of Disease Collaborative Network. Global Burden of Disease Study 2021 (GBD 2021) Results.

[20] Molot J., Sears M., Marshall L.M., Bray R.I. (2021). Neurological susceptibility to environmental exposures: Pathophysiological mechanisms in neurodegeneration and multiple chemical sensitivity. Rev. Environ. Health.

[21] Zhou X., Zhou X., Wang C., Zhou H. (2023). Environmental and human health impacts of volatile organic compounds: A perspective review. Chemosphere.

[22] Mir R.H., Sawhney G., Pottoo F.H., Mohi-Ud-Din R., Madishetti S., Jachak S.M., Ahmed Z., Masoodi M.H. (2020). Role of environmental pollutants in Alzheimer’s disease: A review. Environ. Sci. Pollut. Res. Int..

[23] Hu C.-Y., Fang Y., Li F.L., Dong B., Hua X.G., Jiang W., Zhang H., Lyu Y., Zhang X.J. (2019). Association between ambient air pollution and Parkinson’s disease: Systematic review and meta-analysis. Environ. Res..

[24] Delamarre A., Meissner W.G. (2017). Epidemiology, environmental risk factors and genetics of Parkinson’s disease. Presse Med..

[25] Palacios N., Fitzgerald K.C., Hart J.E., Weisskopf M., Schwarzschild M.A., Ascherio A., Laden F. (2017). Air pollution and risk of Parkinson’s disease in a large prospective study of men. Environ. Health Perspect..

[26] Kioumourtzoglou M.A., Schwartz J.D., Weisskopf M.G., Melly S.J., Wang Y., Dominici F., Zanobetti A. (2016). Long-term PM2.5 exposure and neurological hospital admissions in the northeastern United States. Environ. Health Perspect..

[27] Lee P.C., Liu L.L., Sun Y., Chen Y.A., Liu C.C., Li C.Y., Yu H.L., Ritz B. (2016). Traffic-related air pollution increased the risk of Parkinson’s disease in Taiwan: A nationwide study. Environ. Int..

[28] Liu R., Young M.T., Chen J.C., Kaufman J.D., Chen H. (2016). Ambient air pollution exposures and risk of Parkinson disease. Environ. Health Perspect..

[29] Ritz B., Lee P.C., Hansen J., Lassen C.F., Ketzel M., Sorensen M., Raaschou-Nielsen O. (2016). Traffic-related air pollution and Parkinson’s disease in Denmark: A case-control study. Environ. Health Perspect..

[30] Kirrane E.F., Bowman C., Davis J.A., Hoppin J.A., Blair A., Chen H., Patel M.M., Sandler D.P., Tanner C.M., Vinikoor-Imler L. (2015). Associations of ozone and PM2.5 concentrations with Parkinson’s disease among participants in the agricultural health study. J. Occup. Environ. Med..

[31] Dutheil F., Beaune P., Tzourio C., Loriot M.A., Elbaz A. (2010). Interaction between ABCB1 and professional exposure to organochlorine insecticides in Parkinson disease. Arch. Neurol..

[32] Bharathi I.S.S., Rao K.S. (2007). Copper- and iron-induced differential fibril formation in alpha-synuclein: TEM study. Neurosci. Lett..

[33] Block M.L., Elder A., Auten R.L., Bilbo S.D., Chen H., Chen J.C., Cory-Slechta D.A., Costa D., Diaz-Sanchez D., Dorman D.C. (2012). The outdoor air pollution and brain health workshop. Neurotoxicology.

[34] Hirsch E.C., Hunot S. (2009). Neuroinflammation in Parkinson’s disease: A target for neuroprotection?. Lancet Neurol..

[35] Segalowitz S.J. (2008). Public health, brain health, and the dangers of air pollution for neural development. Brain Cogn..

[36] Kaplin A.I., Williams M. (2007). How common are the “common” neurologic disorders?. Neurology.

[37] Kalenik S., Zaczek A., Rodacka A. (2025). Air pollution-induced neurotoxicity: The relationship between air pollution, epigenetic changes, and neurological disorders. Int. J. Mol. Sci..

[38] Moulton P.V., Yang W. (2012). Air pollution, oxidative stress, and Alzheimer’s disease. J. Environ. Public. Health.

[39] Kim D.-K., Park J.-D., Choi B.-S. (2014). Mercury-induced amyloid-beta (Aβ) accumulation in the brain is mediated by disruption of Aβ transport. J. Toxicol. Sci..

[40] Ballinger S.W. (2021). Mitochondrial dysfunction as a hallmark of environmental injury. Cells.

[41] Huang M., Bargues-Carot A., Riaz Z., Wickham H., Zenitsky G., Jin H., Anantharam V., Kanthasamy A., Kanthasamy A.G. (2022). Impact of Environmental Risk Factors on Mitochondrial Dysfunction, Neuroinflammation, Protein Misfolding, and Oxidative Stress in the Etiopathogenesis of Parkinson’s Disease. Int. J. Mol. Sci..

[42] Calabró V., Garces M., Cáceres L., Magnani N.D., Marchini T., Freire A., Vico T., Martinefski M., Vanasco V., Triodi V. (2021). Urban air pollution induces alterations in redox metabolism and mitochondrial dysfunction in mice brain cortex. Arch. Biochem. Biophys..

[43] Olloquequi J., Díaz-Peña R., Verdaguer E., Ettcheto M., Auladell C., Camins A. (2024). From Inhalation to Neurodegeneration: Air Pollution as a Modifiable Risk Factor for Alzheimer’s Disease. Int. J. Mol. Sci..

[44] Uversky V.N., Li J., Fink A.L. (2001). Pesticides directly accelerate the rate of alpha-synuclein fibril formation: A possible factor in Parkinson’s disease. FEBS Lett..

[45] Uversky V.N., Li J., Fink A.L. (2001). Metal-triggered structural transformations, aggregation, and fibrillation of human α-synuclein: A possible molecular link between Parkinson’s disease and heavy metal exposure. J. Biol. Chem..

[46] Ascherio A., Chen H., Weisskopf M.G., O’Reilly E., McCullough M.L., Calle E.E., Schwarzschild M.A., Thun M.J. (2006). Pesticide exposure and risk for Parkinson’s disease. Ann. Neurol..

[47] Priyadarshi A., Khuder S.A., Schaub E.A., Shrivastava S. (2000). A meta-analysis of Parkinson’s disease and exposure to pesticides. Neurotoxicology.

[48] Corrigan F.M., Wienburg C.L., Shore R.F., Daniel S., Mann D. (2000). Organochlorine insecticides in substantia nigra in Parkinson’s disease. J. Toxicol. Environ. Health A.

[49] Le Couteur D.G., McLean A.J., Taylor M.C., Woodham B.L., Board P.G. (1999). Pesticides and Parkinson’s disease. Biomed. Pharmacother..

[50] Gao H.M., Hong J.S., Zhang W., Liu B. (2003). Synergistic dopaminergic neurotoxicity of the pesticide rotenone and inflammogen lipopolysaccharide: Relevance to the etiology of Parkinson’s disease. J. Neurosci..

[51] Keane P.C., Hanson P.S., Patterson L., Blain P.G., Hepplewhite P., Khundakar A.A., Judge S.J., Kahle P.J., LeBeau F.E.N., Morris C.M. (2019). Trichloroethylene and its metabolite TaClo lead to degeneration of substantia nigra dopaminergic neurones: Effects in wild type and human A30P mutant α-synuclein mice. Neurosci. Lett..

[52] Zeliger H.I. (2013). Exposure to lipophilic chemicals as a cause of neurological impairments, neurodevelopmental disorders and neurodegenerative diseases. Interdiscip. Toxicol..

[53] Guehl D., Bezard E., Dovero S., Boraud T., Bioulac B., Gross C. (1999). Trichloroethylene and parkinsonism: A human and experimental observation. Eur. J. Neurol..

[54] Liu M., Shin E.-J., Dang D.-K., Jin C.-H., Lee P.H., Jeong J.H., Park S.-J., Kim Y.-S., Xing B., Xin T. (2018). Trichloroethylene and Parkinson’s disease: Risk assessment. Mol. Neurobiol..

[55] Reddy N.J., Lewis L.D., Gardner T.B., Osterling W., Eskey C.J., Nierenberg D.W. (2007). Two cases of rapid onset Parkinson’s syndrome following toxic ingestion of ethylene glycol and methanol. Clin. Pharmacol. Ther..

[56] Finkelstein Y., Vardi J. (2002). Progressive parkinsonism in a young experimental physicist following long-term exposure to methanol. Neurotoxicology.

[57] Livingston G., Huntley J., Sommerlad A., Ames D., Ballard C., Banerjee S., Brayne C., Burns A., Cohen-Mansfield J., Cooper C. (2020). Dementia prevention, intervention, and care: 2020 report of the Lancet Commission. Lancet.

[58] Chavan H., Krishnamurthy P. (2012). Polycyclic aromatic hydrocarbons (PAHs) mediate transcriptional activation of the ATP binding cassette transporter ABCB6 gene via the aryl hydrocarbon receptor (AhR). J. Biol. Chem..

[59] Ruder A.M., Hein M.J., Hopf N.B., Waters M.A. (2014). Mortality among 24,865 workers exposed to polychlorinated biphenyls (PCBs) in three electrical capacitor manufacturing plants: A ten-year update. Int. J. Hyg. Environ. Health.

[60] Richardson J.R., Roy A., Shalat S.L., von Stein R.T., Hossain M.M., Buckley B., Gearing M., Levey A.I., German D.C. (2014). Elevated serum pesticide levels and risk for Alzheimer disease. JAMA Neurol..

[61] Kilian J., Kitazawa M. (2018). The emerging risk of exposure to air pollution on cognitive decline and Alzheimer’s disease: Evidence from epidemiological and animal studies. Biomed. J..

[62] Haghani A., Morgan T.E., Forman H.J., Finch C.E. (2020). Air pollution neurotoxicity in the adult brain: Emerging concepts from experimental findings. J. Alzheimers Dis..

[63] Calderon-Garciduenas L., Torres-Jardon R., Kulesza R.J., Mansour Y., González-González L.O., Gónzalez-Maciel A., Reynoso-Robles R., Mukherjee P.S. (2020). Alzheimer disease starts in childhood in polluted Metropolitan Mexico City: A major health crisis in progress. Environ. Res..

[64] Crous-Bou M., Gascon M., Gispert J.D., Cirach M., Sánchez-Benavides G., Falcon C., Arenaza-Urquijo E.M., Gotsens X., Fauria K., Sunyer J. (2020). Impact of urban environmental exposures on cognitive performance and brain structure of healthy individuals at risk for Alzheimer’s dementia. Environ. Int..

[65] Calderón-Garcidueñas L., González-Maciel A., Kulesza R.J., González-González L.O., Reynoso-Robles R., Mukherjee P.S., Torres-Jardón R. (2019). Air Pollution, Combustion and Friction Derived Nanoparticles, and Alzheimer’s Disease in Urban Children and Young Adults. J. Alzheimers Dis..

[66] Tsai T.-L., Lin Y.-T., Hwang B.-F., Nakayama S.F., Tsai C.-H., Sun X.-L., Ma C., Jung C.-R. (2019). Fine particulate matter is a potential determinant of Alzheimer’s disease: A systemic review and meta-analysis. Environ. Res..

[67] Younan D., Petkus A.J., Widaman K.F., Wang X., Casanova R., Espeland M.A., Gatz M., Henderson V.W., Manson J.E., Rapp S.R. (2020). Particulate matter and episodic memory decline mediated by early neuroanatomic biomarkers of Alzheimer’s disease. Brain.

[68] Gong Y., Zhang X., Zhao H., Chang J.Z., Zhan G., Yang M., Yao C., Huanhuan Z., Cunrui H., Zengli Y. (2023). Global ambient particulate matter pollution and neurodegenerative disorders: A systematic review of literature and meta-analysis. Environ. Sci. Pollut. Res..

[69] Medehouenou T.C.M., Ayotte P., Carmichael P.-H., Krööger E., Verreault R., Lindsay J., Dewailly É., Tyas S.L., Bureau A., Laurin D. (2019). Exposure to polychlorinated biphenyls and organochlorine pesticides and risk of dementia, Alzheimer’s disease and cognitive decline in an older population: A prospective analysis from the Canadian Study of Health and Aging. Environ. Health.

[70] Cipriani G., Danti S., Carlesi C., Borin G. (2018). Danger in the air: Air pollution and cognitive dysfunction. Am. J. Alzheimers Dis. Other Demen..

[71] Babic Leko M., Mihelcic M., Jurasovic J., Perković M.N., Španić E., Sekovanić A., Orct T., Zubčić K., Horvat L.L., Pleić N. (2022). Heavy metals and essential metals are associated with cerebrospinal fluid biomarkers of Alzheimer’s disease. Int. J. Mol. Sci..

[72] Huat T.J., Camats-Perna J., Newcombe E.A., Valmas N., Kitazawa M., Medeiros R. (2019). Metal toxicity links to Alzheimer’s disease and neuroinflammation. J. Mol. Biol..

[73] Kukull W.A., Larson E.B., Bowen J.D., McCormick W.C., Teri L., Pfanschmidt M.L., Thompson J.D., O’Meara E.S., Brenner D.E., van Belle G. (1995). Solvent exposure as a risk factor for Alzheimer’s disease: A case-control study. Am. J. Epidemiol..

[74] Ritchie G.D., Still K.R., Alexander W.K., Nordholm A.F., Wilson C.L., Rossi J., Mattie D.R. (2001). A review of the neurotoxicity risk of selected hydrocarbon fuels. J. Toxicol. Environ. Health B Crit. Rev..

[75] Tisch U., Schlesinger I., Ionescu R., Nassar M., Axelrod N., Robertman D., Tessler Y., Azar F., Marmur A., Aharon-Peretz J. (2013). Detection of Alzheimer’s and Parkinson’s disease from exhaled breath using nanomaterial-based sensors. Nanomedicine.

[76] Mazzatenta A., Pokorski M., Sartucci F., Domenici L., Di Giulio C. (2015). Volatile organic compounds (VOCs) fingerprint of Alzheimer’s disease. Respir. Physiol. Neurobiol..

[77] Tiele A., Wicaksono A., Daulton E., Ifeachor E., Eyre V., Clarke S., Timings L., Pearson S., Covington J.A., Li X. (2020). Breath-based non-invasive diagnosis of Alzheimer’s disease: A pilot study. J. Breath Res..

[78] Xiong C., Lu R., Bui Q., Popp B., Schindler S., Shriver L., Cruchaga C., Hassenstab J., Benzinger T., Agboola F. (2025). Person-specific digitally measured air pollutant exposure and biomarkers of Alzheimer disease: Findings from a pilot study. J. Alzheimers Dis..

[79] Matés J.M., Segura J.A., Alonso F.J., Márquez J. (2010). Roles of dioxins and heavy metals in cancer and neurological diseases using ROS-mediated mechanisms. Free Radic. Biol. Med..

[80] Al-Mousa F., Michelangeli F. (2012). Some commonly used brominated flame retardants cause Ca^2+^ ATPase inhibition, beta-amyloid peptide release and apoptosis in SH–SY5Y neuronal cells. PLoS ONE.

[81] Viberg H., Fredriksson A., Eriksson P. (2003). Neonatal exposure to polybrominated diphenyl ether (PBDE 153) disrupts spontaneous behaviour, impairs learning and memory, and decreases hippocampal cholinergic receptors in adult mice. Toxicol. Appl. Pharmacol..

[82] Murata H., Barnhill L.M., Bronstein J.M. (2022). Air pollution and the risk of Parkinson’s disease: A review. Mov. Disord..

[83] Maher B.A., Ahmed I.A., Karloukovski V., MacLaren D.A., Foulds P.G., Allsop D., Mann D.M., Torres-Jardon R., Calderon-Garciduenas L. (2016). Magnetite pollution nanoparticles in the human brain. Proc. Natl. Acad. Sci. USA.

[84] Lodovici M., Bigagli E. (2011). Oxidative stress and air pollution exposure. J. Toxicol..

[85] Subramaniam N.S., Bawden C.S., Waldvogel H., Faull R.M.L., Howarth G.S., Snell R.G. (2018). Emergence of breath testing as a new non-invasive diagnostic modality for neurodegenerative diseases. Brain Res..

[86] Ho E., Galougahi K.K., Liu C.-C., Bhindi R., Figtree G.A. (2013). Biological markers of oxidative stress: Applications to cardiovascular research and practice. Redox Biol..

[87] Capuano R., Ciotti M., Catini A., Bernardini S., Di Natale C. (2024). Clinical applications of volatilomic assays. Crit. Rev. Clin. Lab. Sci..

[88] van Helmond W., van Bochove M.A., van Herwijnen A.W., van Riemsdijk J.J.H., de Poot C.J., de Puit M. (2019). Chemical profiling of fingerprints using mass spectrometry. Forensic Chem..

[89] Henderson B., Slingers G., Pedrotti M., Pugliese G., Malásková M., Bryant L., Lomonaco T., Ghimenti S., Moreno S., Cordell R. (2021). The peppermint breath test benchmark for PTR-MS and SIFT-MS. J. Breath Res..

[90] Blanco F.G., Vidal-de-Miguel G. (2023). Breath Analysis by Secondary Electro-Spray Ionization—Mass Spectrometry to Interrogate Biologically Significant Metabolites Non-Invasively. Crit. Rev. Anal. Chem..

[91] Lau H.-C., Yu J.-B., Lee H.-W., Huh J.-S., Lim J.-O. (2017). Investigation of exhaled breath samples from patients with Alzheimer’s disease using gas chromatography-mass spectrometry and an exhaled breath sensor system. Sensors.

[92] Vasilescu A., Hrinczenko B., Swain G.M., Peteu S.F. (2021). Exhaled breath biomarker sensing. Biosens. Bioelectron..

[93] Zong B., Wu S., Yang Y., Li Q., Tao T., Mao S. (2025). Smart gas sensors: Recent developments and future prospective. Nano-Micro Lett..

[94] Wang C., Li M., Jiang H., Tong H., Feng Y., Wang Y., Pi X., Guo L., Nie M., Feng H. (2016). Comparative analysis of VOCs in exhaled breath of amyotrophic lateral sclerosis and cervical spondylotic myelopathy patients. Sci. Rep..

[95] Feng T. (2023). Applications of artificial intelligence to diagnosis of neurodegenerative diseases. Stud. Health Technol. Inform..

[96] Hakim M., Broza Y.Y., Barash O., Peled P., Phillips M., Amann A., Haick H. (2012). Volatile organic compounds of lung cancer and possible biochemical pathways. Chem. Rev..

[97] Mansurova M., Ebert B.E., Blank L.M., Ibáñez A.J. (2018). A breath of information: The volatilome. Curr. Genet..

[98] Mochalski P., King J., Klieber M., Unterkofler K., Hinterhuber H., Baumann M., Amann A. (2013). Blood and breath levels of selected volatile organic compounds in healthy volunteers. Analyst.

[99] de Lacy Costello B., Amann A., Al-Kateb H., Flynn C., Filipiak W., Khalid T., Osborne D., Ratcliffe N.M. (2014). A review of the volatiles from the healthy human body. J. Breath Res..

[100] Di Natale C., Paolesse R., Martinelli E., Capuano R. (2014). Solid-state gas sensors for breath analysis: A review. Anal. Chim. Acta.

[101] Drabinska N., Flynn C., Ratcliffe N., Belluomo I., Myridakis A., Gould O., Fois M., Smart A., Devine T., De Lacy Costello B. (2021). A literature survey of all volatiles from healthy human breath and bodily fluids: The human volatilome. J. Breath Res..

[102] Gasparri R., Santonico M., Valentini C., Sedda G., Borri A., Petrella F., Maisonneuve P., Pennazza G., D’Amico A., Di Natale C. (2016). Volatile signature for the early diagnosis of lung cancer. J. Breath Res..

[103] Di Gilio A., Palmisani J., Ventrella G., Facchini L., Catino A., Varesano N., Pizzutilo P., Galetta D., Borelli M., Barbieri P. (2020). Breath analysis: Comparison among methodological approaches for breath sampling. Molecules.

[104] Palmisani J., Di Gilio A., Picciariello A., Altomare D.F. (2025). Sampling Technologies. Volatile Organic Compounds.

[105] Miekisch W., Kischkel S., Sawacki A., Liebau T., Mieth M., Schubert J.K. (2008). Impact of sampling procedures on the results of breath analysis. J. Breath. Res..

[106] Amorim L.C.A., Cardeal Z.L. (2007). Breath air analysis and its use as a biomarker in biological monitoring of occupational and environmental exposure to chemical agents. J. Chromatogr. B.

[107] Herbig J., Titzmann T., Beauchamp J., Kohl I., Hansel A. (2008). Buffered end-tidal (BET) sampling—A novel method for real-time breath-gas analysis. J. Breath Res..

[108] Poli D., Goldoni M., Corradi M., Acampa O., Carbognani P., Internullo E., Casalini A., Mutti A. (2010). Determination of aldehydes in exhaled breath of patients with lung cancer by means of on-fiber-derivatisation SPME-GC/MS. J. Chromatogr. B Anal. Technol. Biomed. Life Sci..

[109] Akman H., Bayrakli I., Kutluhan S. (2018). Investigation of Correlation between Neurological Diseases and Breath Hexanal. J. Bioanal. Biomed..

[110] Ratcliffe N., Wieczorek T., Drabinska N. (2020). A mechanistic study and review of volatile products from peroxidation of unsaturated fatty acids: An aid to understanding the origins of volatile organic compounds from the human body. J. Breath Res..

[111] Sutaria S.R., Gori S.S., Morris J.D., Xie Z., Fu X.-A., Nantz M.H. (2022). Lipid Peroxidation Produces a Diverse Mixture of Saturated and Unsaturated Aldehydes in Exhaled Breath That Can Serve as Biomarkers of Lung Cancer—A Review. Metabolites.

[112] Aluf Y., Vaya J., Khatib S., Loboda Y., Kizhner S., Finberg J.P. (2010). Specific oxidative stress profile associated with partial striatal dopaminergic depletion by 6-hydroxydopamine as assessed by a novel multifunctional marker molecule. Free Radic. Res..

[113] Haripriya P., Rangarajan M., Pandya H.J. (2023). Breath VOC analysis and machine learning approaches for disease screening: A review. J. Breath Res..

[114] Bach J.P., Gold M., Mengel D., Hattesohl A., Lubbe D., Schmid S., Tackenberg B., Rieke J., Maddula S., Baumbach J.I. (2015). Measuring compounds in exhaled air to detect Alzheimer’s disease and Parkinson’s disease. PLoS ONE.

[115] Cope K.A., Watson M.T., Foster W.M., Sehnert S.S., Risby T.H. (2004). Effects of ventilation on the collection of exhaled breath in humans. J. Appl. Physiol..

[116] Phillips M., Cataneo R.N., Cummin A.R.C., Gagliardi A.J., Gleeson K., Greenberg J., Maxfield R.A., Rom W.N. (2003). Detection of lung cancer with volatile markers in the breath. Chest.

[117] Alonso M., Sanchez J.M. (2013). Analytical challenges in breath analysis and its application to exposure monitoring. Trends Anal. Chem..

[118] Lourenço C., Turner C. (2014). Breath Analysis in Disease Diagnosis: Methodological Considerations and Applications. Metabolites.

[119] Filipiak W., Ruzsanyi V., Mochalski P., Filipiak A., Bajtarevic A., Ager C., Denz H., Hilbe W., Jamnig H., Hackl M. (2012). Dependence of exhaled breath composition on exogenous factors, smoking habits and exposure to air pollutants. J. Breath Res..

[120] Blanchet L., Smolinska A., Baranska A., Tigchelaar E., Swertz M., Zhernakova A., Dallinga J.W., Wijmenga C., van Schooten F.J. (2017). Factors that influence the volatile organic compound content in human breath. J. Breath Res..

[121] Ubeda C., Vázquez-Carretero M.D., Luque-Tirado A., Ríos-Reina R., Rubio-Sánchez R., Peral M.J. (2023). Fecal volatile organic compounds and microbiota associated with the progression of cognitive impairment in Alzheimer’s disease. Int. J. Mol. Sci..

[122] De Pablo-Fernandez E., Gebeyehu G.G., Flain F., Slater R., Frau A., Ijaz U.Z., Warner T., Probert C. (2022). The faecal metabolome and mycobiome in Parkinson’s disease. Park. Relat. Disord..

[123] Smiełowska M., Ligor T., Kupczyk W., Szeliga J., Jackowski M., Buszewski B. (2023). Screening for volatile biomarkers of colorectal cancer by analyzing breath and fecal samples using thermal desorption combined with GC-MS (TD-GC-MS). J. Breath Res..

[124] Cook S.I., Sellin J.H. (1998). Review article: Short chain fatty acids in health and disease. Aliment. Pharmacol. Ther..

[125] Hamer H.M., Jonkers D., Venema K., Vanhoutvin S., Troost F.J., Brummer R.J. (2008). Review article: The role of butyrate on colonic function. Aliment. Pharmacol. Ther..

[126] Chen H., Meng L., Shen L. (2022). Multiple roles of short-chain fatty acids in Alzheimer disease. Nutrition.

[127] Di Cagno R., De Angelis M., De Pasquale I., Ndagijimana M., Vernocchi P., Ricciuti P., Gagliardi F., Laghi L., Crecchio C., Guerzoni M.E. (2011). Duodenal and faecal microbiota of celiac children: Molecular, phenotype and metabolome characterization. BMC Microbiol..

[128] Nazik H., Yıldız B.T. (2019). Evaluation of skin disorders, skin sebum and moisture in patients with Parkinson’s disease. Neurol. Asia.

[129] Fu W., Linxin X., Qiwen Y., Fang J., Zhao G., Li Y., Pan C., Dong H., Wang D., Ren H. (2022). Artificial intelligent olfactory system for the diagnosis of Parkinson’s disease. ACS Omega.

[130] Sinclair E., Walton-Doyle C., Sarkar D., Hollywood K.A., Milne J., Lim S.H., Kunath T., Rijs A.M., de Bie R.M.A., Silverdale M. (2021). Validating differential volatilome profiles in Parkinson’s disease. ACS Cent. Sci..

[131] Trivedi D.K., Sinclair E., Xu Y., Sarkar D., Walton-Doyle C., Liscio C., Banks P., Milne J., Silverdale M., Kunath T. (2019). Discovery of volatile biomarkers of Parkinson’s disease from sebum. ACS Cent. Sci..

[132] Tsuda T., Nonome T., Goto S., Takeda J.-I., Tsunoda M., Hirayama M., Ohno K. (2019). Application of skin gas GC/MS analysis for prediction of the severity scale of Parkinson’s disease. Chromatography.

[133] Agapiou A., Amann A., Mochalski P., Statheropoulos M., Thomas C. (2015). Trace detection of endogenous human volatile organic compounds for search, rescue and emergency applications. Trends Anal. Chem..

